# 

**Published:** 1883-05

**Authors:** 


MAY, 1883.

NUMBER 10, VOLUME XXII.

### THE BUFFALO Medical and Surgical Journal.

S.G.O. INDEXED

EDITORS:

THOS. LOTHROP, M. D., A. R. DAVIDSON, M. D., P. W. VAN PEYMA, M. D.

BUFFALO:

PUBLISHED BY THE MEDICAL JOURNAL ASSOCIATION.

No. 5 WEST CHIPPEWA ST.

**Read the Notice of this Journal among the Advertisements.**

Entered at the Post-Office at Buffalo, N. Y., as Second-class Mail Matter.

